# How to ensure an appropriate oral health workforce? Modelling future scenarios for the Netherlands

**DOI:** 10.1186/s12960-024-00957-2

**Published:** 2024-11-08

**Authors:** Jip Janssen, Ave Pöld, Md Monirul Islam, Orsolya Németh, Jostein Grytten, Noel Woods, Stefan Listl

**Affiliations:** 1 Department of Dentistry, Radboud University Medical Center, Nijmegen, The Netherlands; 2 Estonian Dental Association, Tallinn, Estonia; 3 Heidelberg Institute of Global Health, Section for Oral Health, Heidelberg University Hospital, Heidelberg University, Heidelberg, Germany; 4 Centre for Policy Studies, Cork University Business School, University College Cork, Cork, Ireland; 5 Department of Public Dental Health, Semmelweis University, Budapest, Hungary; 6 Department of Community Dentistry, University of Oslo, Oslo, Norway

**Keywords:** Oral health, Workforce planning model, Provider supply, Provider requirement, Epidemiological scenarios, Skill-mix, Student intake, Training duration

## Abstract

**Background:**

Current methods for oral health workforce planning lack responsiveness to dynamic needs, hampering efficiency, equity and sustainability. Effective workforce planning is vital for resilient health care systems and achieving universal health coverage. Given this context, we developed and operationalised a needs-adaptive oral health workforce planning model and explored the potential of various future scenarios.

**Methods:**

Using publicly available data, including the Special Eurobarometer 330 Oral Health Survey, we applied the model in a hypothetical context focusing on the Dutch population’s dental needs from 2022 to 2050. We compared current and future provider supply and requirement and examined, in addition to a base case scenario, several alternative scenarios. These included epidemiological transition scenarios with different oral health morbidity trajectories, skill-mix scenarios with independent oral hygienists conducting check-ups and multiple dental student intake and training duration (5 instead of 6 years) scenarios.

**Results:**

Based on the aforementioned historical data, our model projects that provider requirement will exceed supply for the planning period. If the percentage of people having all natural teeth increases by 10% or 20% in 2032, 34 or 68 additional full-time equivalent (FTE) dentists will be required, respectively, compared to the base case scenario. In the skill-mix scenario, the model indicates that prioritising oral hygienists for check-ups and shifting dentists’ focus to primarily complex care could address population needs more efficiently. Among the student intake and training duration scenarios, increasing intake to 375 and, to a lesser extent, reducing training to 5 years is projected to most effectively close the provider gap.

**Conclusions:**

The study underscores the importance of understanding oral health morbidity trajectories for effective capacity planning. Due to limited dental epidemiological data, projections carry substantial uncertainty. Currently, demand for FTE dentists seems to exceed supply, though this may vary with epidemiological changes. Skill-mix strategies could offer efficiency gains by redistributing tasks, while adjustments in dental intake and training duration could also help address the requirement-supply gap. Resolving dentistry workforce challenges requires a multifaceted approach, including strengthening oral epidemiology projections, addressing the root causes of dental health issues and prioritising harmonious dental public health and general practice prevention measures.

**Supplementary Information:**

The online version contains supplementary material available at 10.1186/s12960-024-00957-2.

## Background

As highlighted by the 2021 WHO Resolution on Oral Health, the methods currently used for planning resources and workforce for dental care are largely unresponsive to dynamic changes in people’s needs [1]. This rigidity has detrimental repercussions for oral care financing and blocks potential gains in efficiency, equity and sustainability [2]. The 2022 WHO Global Strategy on Oral Health includes ‘innovative workforce models to respond to population needs for oral health’ as a guiding principle backing the WHO’s vision: universal health coverage (UHC) for dental health for all individuals and communities by 2030, enabling them to enjoy the highest attainable state of oral health and contributing to healthy and productive lives [3].

Effective health workforce planning is pivotal for the viability of health care systems, UHC and achievement of the sustainable development goals [4–6]. A well-thought-out plan ensures the delivery of the right care, in the right place, at the right time, by the right number and mix of skilled professionals, addressing the evolving health needs of populations at an affordable cost [7]. Without successful planning, critical issues may arise, including limited access to services, unmet needs, degradation of care quality, increased risks to patient safety, low staff morale, staff retention concerns and poor stewardship of health care budgets. Workforce expenditures can account for up to 70% of national health system budgets, making effective planning essential for efficient resource allocation and long-term sustainability [8].

The traditional approach to oral health workforce planning emphasises demographic projections and fixed ratios of providers or services to population, neglecting crucial factors such as evolving population characteristics and oral health needs over time [2]. This leads to inefficiencies and disparities in access to dental care. Thus, there is a necessity for a more nuanced and dynamic approach to oral health capacity planning that considers changes in dental health status, enabling more accurate estimates of workforce requirements and better resource allocation. Workforce planning in oral health care is often constrained due to the complexity of projecting future health trends and the availability of reliable, up-to-date data. Assumptions about dental team configurations can substantially influence these projections, which are further complicated by regional disparities in workforce distribution. These limitations can lead to inaccuracies in projecting workforce needs and hinder the development of responsive, effective policy solutions.

In dentistry, ‘skill-mix’ refers to a framework where the entire clinical team participates in delivering service activities, determined by their level of education, training and scope of practice [9–12]. The pros and cons of skill-mix in the health workforce have been explored in general medicine, suggesting increased cost-effectiveness, sustained service delivery quality and enhanced health outcomes [12, 13]. Research in the oral health context also suggests that incorporating diverse skills in dental practice can enhance efficiency, service effectiveness and workforce capacity [9, 14–16]. However, previous attempts to implement skill-mix approaches in the dental field have demonstrated some complexities, highlighting the need for further exploration and careful consideration in planning [9].

The aim of this study was to develop and operationalise an oral health workforce planning model to compare the current and projected provider supply and requirement of dental care providers in the Netherlands and explore the potential effects of changes in oral health status, skill-mix and student intake and training duration.

## Methods

**Fig. 1:**
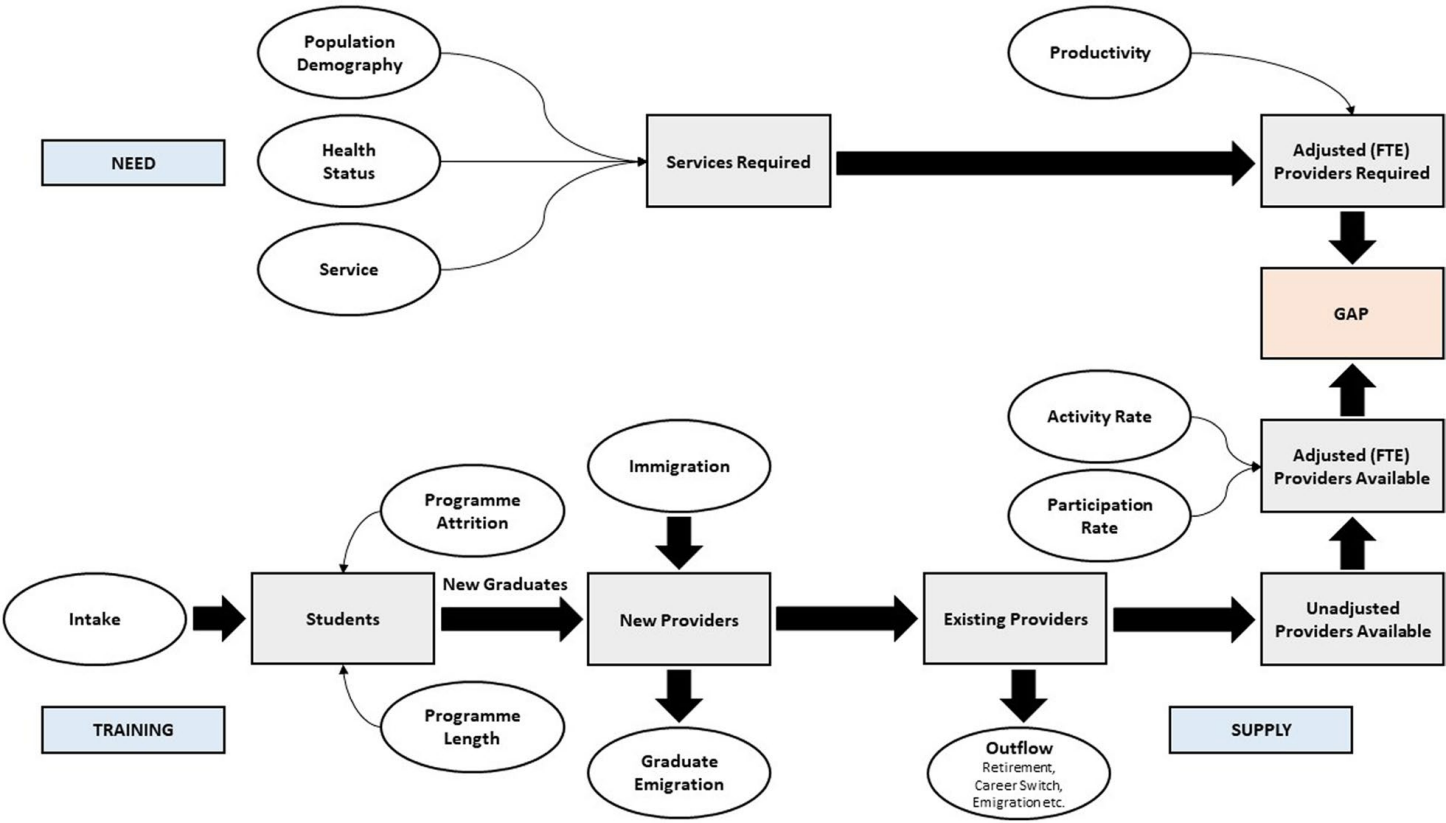
Graphical representation of the needs-adaptive workforce planning framework (adapted from Tomblin Murphy et al. [20]). FTE: full-time equivalent

This study is part of the PRUDENT project (Prioritization, incentives and Resource use for sUstainable DENTistry), funded by the European Union’s Horizon Europe research and innovation programme [17]. Partners include Denmark, Estonia, France, Germany, Hungary, Ireland, Malta, the Netherlands, Norway, Portugal and the United Kingdom. One objective is to build a needs-adaptive oral health workforce planning model that explicitly accounts for public health needs, demographic changes and skill-mix. Building upon a framework of linked spreadsheets developed in previous work by two current authors (Ahern et al.), we created a more comprehensive model in Microsoft Excel that includes options for evaluating dental need and skill-mix scenarios [18, 19]. In addition, our model incorporates a module for exploring student intake and training duration scenarios. The conceptual framework underpinning our model, depicted in Fig. 1, consists of two main components: provider supply (availability) and provider requirement. A description of both components, the explored hypothetical scenarios and the model’s application is provided below.

### Provider supply

The model’s annual provider supply component consists of (i) existing stock, (ii) flow and (iii) newly trained. Existing stock is the current supply of licenced dentists. Flow is broken down into inflow and outflow. Inflow includes new practitioners, excluding those newly qualified in the country of study. In our study, inflow consists of those trained abroad and immigrating to the Netherlands to practice. Outflow includes those retiring, changing careers, emigrating, taking a break or becoming incapacitated. Flow is obtained by subtracting outflow from inflow. The number of newly trained practitioners available to work in the Netherlands is calculated in two steps. First, the number of first-year bachelor dentistry students in the country (intake) is adjusted for attrition using the completion rate as determined by the Dutch Advisory Committee on Medical Manpower Planning (ACMMP) [21]. The completion rate is the percentage of students per cohort that completes the dental training (Bachelor’s and Master’s programmes) at any time, including those with delays. Second, the number of graduates is adjusted using the ACMMP’s graduate retention rate: the proportion of trained dentists who enter and remain in the Dutch workforce [21]. This provides the number of graduates available to work as dental professionals in the Netherlands.

The existing stock of practitioners at the end of the previous year is adjusted for inflow, outflow and new graduates to estimate the provider supply at the end of the current year. This estimate becomes the starting stock figure for the next year, and the procedure is repeated for each year of the model’s planning period, generating stock figures at the end of each year and the start of the next.

To report actual provider supply, stock figures are adjusted for participation rate, time devoted to patient-related activities (billable time) and activity rate. Dental practitioner registers might include those not actively practising, such as professionals in full-time academic positions. We correct for this using a participation rate. Practitioners also engage in non-patient-related activities, including business administration or further training, so we adjust workforce supply based on time devoted to patient-related activities. Finally, as not all practitioners work full-time, their full-time equivalent (FTE) or activity rate is included in the calculations. Altogether, this produces a provider supply figure reported as an FTE number of practising providers engaged in patient-related activities.

### Provider requirement

The annual provider requirement component of the model is broken down into (i) demography, (ii) health status and (iii) service. To incorporate reflections of dental need, based on estimates of oral health status and service utilisation, and to allow for replication by other countries, we used the Special Eurobarometer 330 Oral Health Survey data set, which contains relevant data on all three subcomponents [22]. This survey, conducted in 2009, describes several oral health indicators and covers the populations aged 15 and above in the EU Member States. The following Eurobarometer variables are used in the model: gender and age (demography), number of natural teeth and whether someone had food/pain problems (health status), last dentist visit (service level), number of dentist visits in the past year (service frequency) and reason for last visit (service type).

To determine provider requirement, the model estimates the number of FTE practitioners necessary to address the oral health needs of the population aged 15 and above. This involved categorising individuals into four gender-specific age cohorts: 15–44, 45–64, 65–74 and ≥ 75 years. Using sample service frequency data, we established the total number of visits by gender, age cohort and health status. Total number of visits was then broken down by service type following the Eurobarometer’s subdivision: check-up, routine treatment or emergency treatment. By applying a time component to each service type and multiplying it by the total number of visits, we computed the overall service requirement in hours. This total provider requirement in hours was converted into an equivalent number of FTE practitioners by dividing it by the total hours devoted to patient-related activities by a full-time practitioner per year.

### Scenarios

#### Base case scenario

In this scenario, future annual provider supply and requirement are estimated assuming a continuation of the status quo during the complete model’s planning period, with an unchanging oral epidemiological profile, no skill-mix implementation and the same student intake and training duration.

#### Epidemiological transition scenarios

We recognise that health status by gender and age cohort will, in reality, not remain constant throughout the planning period. While we cannot forecast all possible alterations in oral morbidity, we attempted to project the effects of two conceivable transitions in future health status on FTE provider requirement. Specifically, we developed scenarios for 2032 (10 years into the planning period), increasing the percentage of people having all natural teeth by 10% or 20% across all age cohorts, while adjusting the percentages of individuals without all natural teeth (either ≥ 20 but not all, 10–19, 1–9 or none) proportionally to maintain constant cohort sizes, reflecting assumed intensified preventive policies and care. Here, we assumed no changes in the underlying data regarding food/pain problems, service frequency and service type.

#### Skill-mix scenarios

Assuming that all dental services for primary care needs in our model (check-up, routine treatment or emergency treatment) are delivered by dentists only does not reflect reality [19]. Therefore, we included skill-mix implementation, assuming delegation of tasks to oral hygienists. In the Netherlands, oral hygienists play a key role in maintaining and promoting oral health through preventive care, teeth cleaning, periodontal care, X-rays and patient education. They work closely with dentists under direct supervision but also have substantial autonomy in their clinical activities. We evaluated the effects on FTE provider requirement of a scenario where, in 2032, oral hygienists independently conduct all check-ups, without dentist supervision. This builds on an ongoing experiment in the Netherlands where participating oral hygienists independently treat primary cavities, administer local anaesthesia and take and assess X-rays [23–25]. We hypothesised that, as a result of the assumed skill-mix application in 2032, time required per check-up might decrease due to greater efficiency and streamlining as this becomes the core task for oral hygienists, while realising that various factors, including patient complexity, staff skills and experience, continue to influence treatment timings [26]. We therefore also analysed the potential impact of reducing check-up times by 5 min on provider requirement.

#### Student intake and training duration scenarios

In Dutch dentistry, a substantial number of dentists is expected to retire soon due to the ageing occupational group. In 10 years, 42% of currently practicing dentists will likely have ceased their practice [21]. The ACMMP, which advises on the necessary capacity and distribution of health care professionals in the Netherlands, recommends increasing the annual intake of first-year bachelor dentistry students to preferably 375, or at least 345, to balance demand for and supply of dentists over time. In 2022, the intake was 261 [21]. The Dutch cabinet plans to gradually adhere to at least the minimum intake advise of 345 [27]. However, due to funding constraints, the cabinet is considering making the required additional budget for raising the intake (partly) available by reducing dental training duration from 6 to 5 years [27, 28]. If this reduction is enacted and executed, the yearly intake could gradually increase from 2025 onwards [27].

Six scenarios, presented in Table 1, were run to explore the effects of different combinations of student intake and training duration from 2025 onwards, including scenarios with no changes to intake and/or training duration, as well as scenarios with increased intakes and a reduction in training duration by 1 year.

**Table 1:** Student intake and training duration: six scenarios

Scenario	Annual dentistry student intake (*n*)^a^	Training duration (years)
Scenario 1A (S1A)^b^ Scenario 1B (S1B)	No increase, 261 in 2022–2050 No increase, 261 in 2022–2050	No change: 6 6 until 2024, 5 from 2025 onwards
Scenario 2A (S2A) Scenario 2B (S2B)	261 in 2022–2024, 261 to 345 in 2025–2027, 345 in 2028–2050 261 in 2022–2024, 261 to 345 in 2025–2027, 345 in 2028–2050	No change: 6 6 until 2024, 5 from 2025 onwards
Scenario 3A (S3A) Scenario 3B (S3B)	261 in 2022–2024, 261 to 375 in 2025–2031, 375 in 2032–2050 261 in 2022–2024, 261 to 375 in 2025–2031, 375 in 2032–2050	No change: 6 6 until 2024, 5 from 2025 onwards

^a^Intake includes all first-year bachelor dentistry students in the Netherlands. All intake increases (e.g., 261 to 345) are assumed to be implemented gradually, with equal annual increments

^b^Scenario 1A corresponds to the base case scenario

### Application of the model

To operationalise the model and demonstrate its practicality, we applied it in a hypothetical context, focusing on the dental needs of a population aged 15 and above (aligning it with the Eurobarometer 330 Oral Health data set’s scope, which starts at this age). We used publicly available Dutch data where possible. When such data were lacking, we made assumptions for our inputs. The modelled workforce planning period spans from 2022 to 2050.

To estimate dentist supply, we began with the existing dentist stock at the end of 2021 from a survey by Regioplan Policy Research, excluding oral and maxillofacial surgeons and orthodontists [29]. Inflow data and assumptions about future inflow relied on ACMMP’s 2024–2027 capacity plan [21]. Regarding outflow, a previous ACMMP calculation suggests that around 66% is age-related (retirement), while 33% is attributed to other factors, assuming a combination of career changes, emigration, taking a break and work incapacity [30]. By integrating these proportions with ACMMP future outflow estimates, we calculated annual outflow percentages for the planning period [21]. Relevant data for 2042–2050 being unavailable, we applied the average percentage used for 2022–2041. Data on annual student intake and completion and graduate retention rates were obtained from ACMMP and Regioplan Policy Research reports [21, 29–31]. Assumptions about newly trained in future years were also based on these figures. A participation rate of 95% and a time allocation of 82% to patient-related activities were used, based on previous studies [19, 29]. Dentists were estimated to spend 85% of their time with patients aged 15 and above, based on 2021 dentist visit and population data [32–34]. The applied average activity rate of dentists was 0.86 FTE (1 FTE equalling 40 h) [29]. Regarding the skill-mix scenarios, we assumed a time allocation of 86% to patient-related tasks for oral hygienists, based on survey data, and that, like dentists, they spend 85% of their time with patients aged 15 and above [29]. These figures were assumed to remain constant during the planning period.

To estimate dentist requirement, we analysed, using IBM SPSS Statistics 29, the Dutch sample data from the Eurobarometer 330 Oral Health data set as described above [35]. The sample data were then applied to both current population data and projections published by Statistics Netherlands to simulate the total provider requirement for the planning period [32, 36, 37].

## Results

### Base case scenario

In 2022, there were an estimated 10,156 licensed dentists in the Netherlands. The model indicates that 5783 FTEs provided patient-related dental services to the population aged 15 and above in 2022 (provider supply), accounting for annual flow, newly trained, participation rate, time devoted to patient-related activities and activity rate.

By combining 2022 population statistics for individuals aged 15 and above with data on dental service frequency and type from the Dutch Eurobarometer 330 Oral Health data set (provided in Table 2), we calculated the total hours required of FTE dentists in that year. Here, we applied specific time components to each service type, assuming 20, 30 and 40 min per check-up, routine and emergency treatment, respectively [19]. Assuming that FTE dentists work 1271 h annually (equivalent to 45.6 working weeks, 40 h worked per week and a time allocation of 82% to patient-related activities of which 85% with patients aged 15 and above), the model produces an FTE provider requirement figure of 7073 dentists. Comparing this with the FTE provider supply figure of 5783, the model suggests that in 2022, provider requirement exceeded supply by 1.2 times.

**Table 2:** Type of dental visits by age cohort and gender in a 12-month period (rounded)

Age cohort	Gender	People (*n*)	Visits (*n*)	Service type					
				Check-up (*n*)	%	Routine (*n*)	%	Emergency (*n*)	%
15–44 years	Male Female	3,321,000 3,237,000	5,588,000 5,514,000	4,283,000 4,551,000	77 83	802,000 568,000	14 10	502,000 395,000	9 7
45–64 years	Male Female	2,395,000 2,400,000	3,757,000 4,408,000	2,508,000 3,634,000	67 82	760,000 581,000	20 13	489,000 194,000	13 4
65–74 years	Male Female	962,000 997,000	1,430,000 1,227,000	992,000 1,012,000	69 82	246,000 61,000	17 5	192,000 155,000	13 13
≥ 75 years	Male Female	678,000 889,000	678,000 603,000	370,000 492,000	55 82	166,000 0	24 0	143,000 111,000	21 18
Total		14,878,000	23,205,000	17,842,000	77	3,184,000	14	2,180,000	9

Estimates obtained by combining 2022 population statistics for individuals aged 15 and above with data on dental service frequency and type from the Dutch Eurobarometer 330 Oral Health data set

**Fig. 2:**
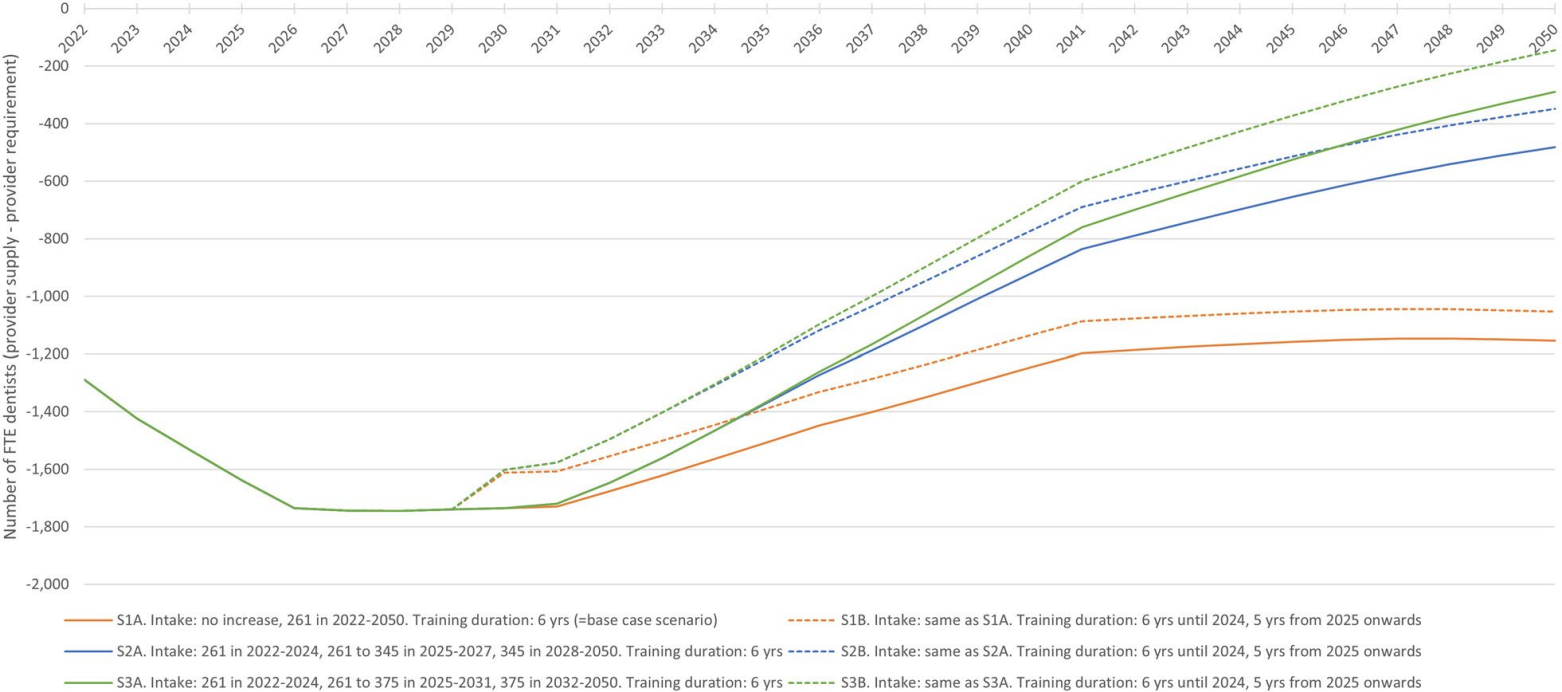
Base case and student intake and training duration scenarios results, 2022–2050. Annual number of FTE dentists after subtracting provider requirement from provider supply for six scenarios with variations in yearly intake (number of first-year bachelor dentistry students) and training length. FTE: full-time equivalent

Scenario 1A (S1A) in Fig. 2 shows the base case scenario results for the 2022–2050 period, assuming a continuation of the status quo. It indicates the annual number of FTE dentists after subtracting provider requirement from provider supply, ranging from − 1289 in 2022 to − 1154 in 2050.

### Epidemiological transition scenarios

Table 3 displays the results of the epidemiological transition scenarios where the percentage of people having all natural teeth is increased by either 10% or 20% across all age cohorts in 2032. With a 10% increase, the model indicates that 34 (7493–7459) more FTE dentists will be required compared to the base case scenario for that year. With a 20% increase, this rises to 68 (7527–7459) more FTE dentists.

**Table 3:** Epidemiological transition and skill-mix scenarios results, 2032

	Skill-mix scenarios			
	Base case scenario: no skill-mix. All services conducted by dentists	Skill-mix implemented. All check-ups conducted by oral hygienists, all routine and emergency treatments by dentists		
	Required dentists (FTE)	Required dentists (FTE)	Required oral hygienists (FTE)	Required oral hygienists, check-up times reduced (FTE)^a^
Epidemiological transition scenarios				
Base case scenario: people having all natural teeth remains constant	7459	2539	4691	3518
People having all natural teeth increases by 10%^b^	7493	2415	4841	3631
People having all natural teeth increases by 20%^b^	7527	2292	4991	3744

^a^Here, we assumed that skill-mix implementation leads to a 5-min reduction in check-up times due to greater efficiency among oral hygienists, from 20 to 15 min

^b^Across all age cohorts, while adjusting the percentages of individuals without all natural teeth (either ≥ 20 but not all, 10–19, 1–9 or none) proportionally to maintain constant cohort sizes. FTE: full-time equivalent

An additional epidemiological transition scenario and two robustness checks are provided in Additional file 1.

### Skill-mix scenarios

Table 3 also presents the results of the scenario where oral hygienists independently conduct all check-ups in 2032. The model projects 4920 (7459–2539) fewer required FTE dentists compared to the base case scenario, with 4691 FTE oral hygienists needed. Combining dentists and oral hygienists, the overall FTE provider requirement is projected at 7230 (2539 + 4691), 229 (7459–7230) lower than the base case scenario. According to the model, a 5-min reduction in check-up times for oral hygienists results in 1173 (4691–3518) fewer required FTE oral hygienists and an overall FTE provider requirement figure of 6057 (2539 + 3518), 1402 (7459–6057) lower than the base case scenario.

### Student intake and training duration scenarios

Figure 2 displays the outcomes of the six scenarios on student intake and training duration, with variations in yearly intake and training length. All scenarios project only negative annual FTE dentist values throughout the planning period, indicating that provider requirement exceeds supply, all reaching their lowest point of − 1745 FTE dentists in 2028. From then on, scenario 3B (S3B) yields the smallest negative values, reaching − 144 FTE dentists in 2050.

## Discussion

Our needs-adaptive oral health workforce planning model suggests that, under the base case scenario, for the Dutch population aged 15 and above, provider requirement will be higher than provider supply for the entire 2022–2050 planning period. If, in 2032, the percentage of people that have all natural teeth increases by either 10% or 20%, the model indicates that 34 or 68 more FTE dentists will be required, respectively, when compared to the base case scenario for that same year. Improved preservation of all natural teeth is projected to increase total time required of FTE dentists, as the anticipated rise in check-ups will outweigh the drop in routine and emergency treatments. The skill-mix scenario suggests that population oral health needs could be addressed more efficiently in terms of overall FTEs if check-ups are performed primarily by oral hygienists while reorienting the focus of dentists on more complex oral health care (routine and emergency treatments). If skill-mix implementation leads to a 5-min reduction in check-up times due to greater efficiency among oral hygienists, the model projects a substantial reduction in FTE provider requirement for this staff type. Among the student intake and training duration scenarios, combining the recommended intake of 375 students with a shortened dental training of 5 years is expected to most effectively address the gap between provider requirement and supply over the planning period. Reducing the training duration has a smaller impact on narrowing the provider gap compared to increasing the intake and results in additional newly trained practitioners only once (in 2030, as both the 2024 and 2025 cohorts will graduate in that year). However, the primary aim of the Dutch cabinet’s proposal to reduce training duration is to free up budget for expanding intake, not to close the requirement-supply gap. If the cabinet decides to enact the training shortening, careful consideration should first be given to ensuring quality education, including adequate clinical experience and required competence, as well as potential capacity building and infrastructural changes. Moreover, evaluating the actual cost savings of shortening the training would be essential.

Dentist requirement appears to outweigh supply in the Netherlands, leading to challenges in providing timely dental care [38, 39]. Today’s gap is attributed to factors such as a growing and aging population, an increasing number of people preserving their natural teeth and challenges in attracting and retaining dental professionals. Recent calculations suggest that more dentists are retiring than graduating [21]. Besides increasing provider supply, another policy option is reducing the time practitioners spend on non-patient-related activities [27]. In 2021, dentists spent, on average, 18% of their total working time on non-billable business activities [29]. For oral hygienists, this number was 14%. Reducing administrative burdens, appointing additional administrative personnel or simplifying the use of dental electronic health records could allow dentists and oral hygienists to spend more time on treating patients and reduce unmet need.

Our findings support the need for a shift from a repair-focused approach to dental care to a preventive one, as emphasized in earlier literature [40–42]. To date, dental service delivery remains centered on repair rather than prevention, which also reflects how funding methods shape clinical activities [43]. Furthermore, previous research concluded that increasing the dental workforce alone, without considering population deprivation, would not improve oral health outcomes, leading to the proposal of a contract management system for funding general dental services that incorporates deprivation status [44, 45].

A Cochrane review comparing 24-month with 6-month check-up recall intervals for adults in primary care settings shows moderate to high-certainty evidence of little to no difference in the number of tooth surfaces with caries, gingival bleeding and oral health-related quality of life over a 4-year period [46]. Our model’s data indicate that individuals who visited a dentist within the past year have, on average, approximately two dentist visits per year, suggesting a 6-month recall interval. Here, we could not distinguish between visits for treatment and check-ups. When approximating a 12-month and 24-month check-up recall interval in our model, by dividing the total number of check-ups in 2022 by two and four respectively, we found that provider requirement was now lower than provider supply for both intervals in 2022. This finding could provide a useful groundwork for determining the optimal recall interval for dental check-ups.

A key strength of the study is the extensive collection of existing publicly available data synthesised into a single model, enabling in-depth analyses and yielding new insights and projections. The model’s versatility and flexibility allow for straightforward incorporation of newly available data and convenient implementation of extensions and updates. Our model-based approach, including options for evaluating various scenarios, addresses a gap in workforce planning estimates for the Netherlands and could serve as a starting point for similar case studies in other countries seeking more evidence to inform their oral health strategies [8, 47]. Findings from such models can support policymaking processes, customise investments in the oral health workforce and aid in effective workforce planning within the oral health field and beyond.

Our study is not without limitations. We acknowledge that several assumptions were made to operationalise our model. First, we were unable to project all potential changes in future population oral health status (number of natural teeth and food/pain problems). Apart from the scenarios regarding increased preservation of all natural teeth, we assumed oral morbidity by gender and age cohort to remain constant, which may lead to an overestimation of provider requirement [19]. That is, our model does not factor in potential improvements in health status over time resulting from health workforce planning aimed at meeting needs. Such improvements could reduce the overall necessity for dental services and provider requirement. Second, we also could not model all feasible options of skill-mix implementation, though the model can be adapted for different skill-mix configurations where certain services are provided by (a combination of) alternative providers. Naturally, the effectiveness of these configurations depends on the availability of all involved professionals, as shortages or imbalances could limit the potential benefits of task reallocation. Third, gaps in available and up-to-date oral health data remain a pressing issue, also for operationalising our model. While details for provider supply were mostly available, recent data on provider requirement were largely lacking. For the latter, apart from up-to-date population statistics, we relied on the 2009 Dutch Eurobarometer 330 Oral Health Survey as this was, according to us, still the most comprehensive source to date. The lack of routine collection of relevant and reliable data will continue to hinder effective workforce planning until properly addressed [8, 19, 47–50]. The recent launch of the Dutch Ministry of Health’s ‘Oral Health monitor’ is a welcome first step as it aims to address this issue by inciting new data collection and sharing initiatives, showing trends over time and supporting sound policymaking [51, 52]. Finally, current simulations provide projections only on an aggregate level for the entire population, despite considerable regional differences in the dental workforce within the Netherlands [21, 53, 54]. Regionally sensitive workforce simulations and policy instruments, such as incentivising work in remote areas and regulating market access in cities, thus seem sensible to consider.

## Conclusions

Effective capacity planning in dentistry hinges on understanding oral health morbidity trajectories. In the absence of comprehensive data on dental epidemiology, any projection carries a substantial risk of uncertainty, relying on historical patterns of service delivery. Our model indicates that demand for FTE dentists surpasses supply under current standards of care. However, the dynamics of potential epidemiological changes could lead to varying outcomes, with the future provider supply either falling short, meeting or exceeding the required numbers. Skill-mix implementation presents an opportunity for efficiency improvements in dental care delivery by redistributing tasks and responsibilities between dentists and oral hygienists. Increasing student intake and reducing dental curriculum length are both also expected to help close the provider gap, with the former anticipated to have a greater impact. However, these adjustments alone might not fully address the workforce challenges in dentistry and should first be critically evaluated for feasibility and desirability. Enhancing oral epidemiology projections, addressing the root causes of dental health issues and investing in public health prevention initiatives could offer stronger opportunities for efficiency improvement in oral care delivery, thereby reducing overall demand for services and alleviating workforce pressure.

## Supplementary Information

Supplementary Material 1. Additional epidemiological transition scenario and robustness checks. This file includes an additional epidemiological transition scenario that projects the effects on FTE provider requirement of increasing the percentage of people having 20 or more (but not all) natural teeth by 20%. Additional file 1 also includes two robustness checks. Robustness check 1 explores the effects on provider requirement if 50% of all check-ups are conducted by oral hygienists, rather than full delegation. Robustness check 2 evaluates the implications for oral hygienist requirement if skill-mix implementation leads to a 3-min or 1-min reduction in check-up times (instead of 5 min).

## Data Availability

The data sets analysed during the current study are available from the GESIS Leibniz Institute for the Social Sciences at 10.4232/1.11140 and Statistics Netherlands at https://opendata.cbs.nl/statline/#/CBS/nl/navigatieScherm/thema?themaNr=3600 [22, 32, 36, 37].

## Abbreviations

ACMMP: Advisory Committee on Medical Manpower Planning
FTE: Full-time equivalent
PRUDENT: Prioritization, incentives and Resource use for sUstainable DENTistry
UHC: Universal health coverage
