# Supplementary material for: How to ensure an appropriate oral health workforce? Modelling future scenarios for the Netherlands
Source: Hum Resour Health. 2024 Nov 8;22:73. doi: 10.1186/s12960-024-00957-2 (PMC11549858; doi:10.1186/s12960-024-00957-2)
Supplement: Supplementary file 1 — Supplementary Material 1. Additional epidemiological transition scenario and robustness checks. This file includes an additional epidemiological transition scenario that projects the effects on FTE provider requirement of increasing the percentage of people having 20 or more (but not all) natural teeth by 20%. Additional file 1 also includes two robustness checks. Robustness check 1 explores the effects on provider requirement if 50% of all check-ups are conducted by oral hygienists, rather than full delegation. Robustness check 2 evaluates the implications for oral hygienist requirement if skill-mix implementation leads to a 3-min or 1-min reduction in check-up times (instead of 5 min). [file 12960_2024_957_MOESM1_ESM.pdf]

ADDITIONAL FILE 1

Additional epidemiological transition scenario

Since the loss of one or a few teeth may not always be linked to a focus on prevention (e.g., trauma or orthodontic removal), we explored an additional scenario. This scenario projects the effects on FTE provider requirement of increasing the percentage of people having 20 or more (but not all) natural teeth by 20% (or the maximum feasible increase, if 20% was not possible) across all age cohorts in 2032. The percentage of people having all natural teeth was kept unchanged, while the percentages of individuals with fewer than 20 natural teeth (either 10-19, 1-9 or none) were adjusted proportionally to maintain constant cohort sizes. This scenario aims to further uncover the nuanced relationship between preventive care efforts, tooth preservation and dental service needs.

With a 20% increase in the percentage of people having 20 or more (but not all) natural teeth, the model projects that 7805 FTE dentists will be required, which is 278 (7805-7527) more than the original epidemiological transition scenario, where the percentage of people having all natural teeth is increased by 20%, as shown in Table 3 of the manuscript. The increase in those having 20 or more (but not all) natural teeth thus leads to more time required of FTE dentists. This is also reflected in a rise in dental service frequencies for all types (check-up, routine treatment and emergency treatment), unlike the original scenario described in manuscript, which indicated an increase in check-ups but a decline in routine and emergency treatments.

Robustness check 1

Robustness check 1 explores the effects on provider requirement if, in 2032, 50% of all check-ups are conducted by oral hygienists, rather than full delegation. Table A presents a portion of the original skill-mix scenario results as shown in Table 3 of the manuscript (with 100% of check-ups conducted by oral hygienists). Table B displays the results of robustness check 1, with changes to the original scenario highlighted in red.

Table A. Original skill-mix scenario results (100% of check-ups conducted by oral hygienists), 2032

		**Skill-mix scenarios**		
		Base case scenario: no skill-mix. All services conducted by dentists	Skill-mix implemented. All check-ups conducted by oral hygienists, all routine and emergency treatments by dentists	
		Required dentists (FTE)	Required dentists (FTE)	Required oral hygienists (FTE)
**Epidemiological transition scenarios**	Base case scenario: people having all natural teeth remains constant	7459	2539	4691

Presents a portion of the original skill-mix scenario results as shown in Table 3 of the manuscript, no changes.

Table B. Robustness check 1 results (50% of check-ups conducted by oral hygienists), 2032

		Skill-mix scenarios		
		Base case scenario: no skill-mix. All services conducted by dentists	Skill-mix implemented. 50% of check-ups conducted by oral hygienists. Remaining 50% of check-ups, all routine and emergency treatments conducted by dentists	
		Required dentists (FTE)	Required dentists (FTE)	Required oral hygienists (FTE)
**Epidemiological transition scenarios**	Base case scenario: people having all natural teeth remains constant	7459	4,999	2,346

Presents a portion of the original skill-mix scenario results as shown in Table 3 of the manuscript, with changes to the original scenario highlighted in red.

According to Table B, for the 50% robustness check, the model projects 2460 (7459-4999) fewer required FTE dentists compared to the base case scenario, with 2346 FTE oral hygienists needed. Combining dentists and oral hygienists, the overall FTE provider requirement is projected at 7345 (4999+2346), 114 (7459-7345) lower than the base case scenario.

The 50% robustness check results in an overall FTE provider requirement figure that is 115 (7345-7230) higher than the original skill-mix scenario presented in the manuscript. Additionally, unlike the original skill-mix scenario, this robustness check indicates a greater requirement for dentists than for oral hygienists.

Thus, in a scenario where 50% of check-ups are conducted by oral hygienists rather than full delegation, the model still suggests that population oral health needs could be addressed more efficiently in terms of overall FTEs compared to no skill-mix implementation (albeit to a lesser extent than with a more extensive role substitution).

Robustness check 2

Robustness check 2 evaluates the implications for oral hygienist requirement if, in 2032, skill-mix implementation (assuming 100% of check-ups are conducted by oral hygienists) leads to a 3-minute or 1-minute reduction in check-up times (instead of 5 minutes). The initial estimate of a 5-minute reduction in check-up time, as described in the manuscript, was an assumption made to illustrate the potential impacts associated with task delegation to oral hygienists. This estimate is intended to serve as a hypothetical scenario for consideration rather than a definitive measurement. Our goal was to highlight the possible efficiencies that could be achieved through skill-mix implementation. While we recognize that actual outcomes may vary, we believe that exploring such assumptions can provide valuable insights for policymaking in workforce planning and resource allocation in oral health care.

Table C, which presents a portion of the original skill-mix scenario results as shown in Table 3 of the manuscript, displays the results of robustness check 2, with changes to the original scenario highlighted in red.

Table C. Robustness check 2 results (3- and 1-minute reduction in oral hygienist check-up times), 2032

		Skill-mix scenarios					
		Base case scenario: no skill-mix. All services conducted by dentists	Skill-mix implemented. All check-ups conducted by oral hygienists, all routine and emergency treatments by dentists				
		Required dentists (FTE)	Required dentists (FTE)	Required oral hygienists (FTE)	Required oral hygienists, check-up times reduced (FTE)		
					5-minute reduction (original)	3-minute reduction	1-minute reduction
**Epidemiological transition scenarios**	Base case scenario: people having all natural teeth remains constant	7459	2539	4691	3518	3988	4457

Presents a portion of the original skill-mix scenario results as shown in Table 3 of the manuscript, with changes to the original scenario highlighted in red.

According to our model and as shown in Table C, a 3-minute and 1-minute reduction in check-up times still results in fewer required FTE oral hygienists (3988 for a 3-minute reduction and 4457 for a 1-minute reduction) compared to no reduction in check-up time (4691 FTE), albeit to a lesser extent.
